# Tracking Fish Abundance by Underwater Image Recognition

**DOI:** 10.1038/s41598-018-32089-8

**Published:** 2018-09-13

**Authors:** Simone Marini, Emanuela Fanelli, Valerio Sbragaglia, Ernesto Azzurro, Joaquin Del Rio Fernandez, Jacopo Aguzzi

**Affiliations:** 1 National Research Council of Italy, Institute of Marine Sciences, Forte Santa Teresa, 19032 La Spezia, Italy; 2 Department of Life and Environmental Sciences, Polytechnic University of Marche, Via Brecce Bianche, Ancona, Italy; 3 Department of Biology and Ecology of Fishes, Leibniz-Institute of Freshwater Ecology and Inland Fisheries, Müggelseedamm 310, Berlin, 12587 Germany; 4 Institute for Environmental Protection and Research (ISPRA), Livorno, Italy; 5 Zoological Station A. Dohrn, Villa comunale, Napoli, Italy; 6 Sarti Research Group. Electronics Deparment. Universitat Politecnica de Catalunya (UPC) - Rambla de l’exposició 24, 08800 Vilanova i la Geltrú, Spain; 7 Instituto de Ciencias del Mar (ICM-CSIC). Paseo Mar´ıtimo de la Barceloneta, 37-49. 08003, Barcelona, Spain

## Abstract

Marine cabled video-observatories allow the non-destructive sampling of species at frequencies and durations that have never been attained before. Nevertheless, the lack of appropriate methods to automatically process video imagery limits this technology for the purposes of ecosystem monitoring. Automation is a prerequisite to deal with the huge quantities of video footage captured by cameras, which can then transform these devices into true autonomous sensors. In this study, we have developed a novel methodology that is based on genetic programming for content-based image analysis. Our aim was to capture the temporal dynamics of fish abundance. We processed more than 20,000 images that were acquired in a challenging real-world coastal scenario at the OBSEA-EMSO testing-site. The images were collected at 30-min. frequency, continuously for two years, over day and night. The highly variable environmental conditions allowed us to test the effectiveness of our approach under changing light radiation, water turbidity, background confusion, and bio-fouling growth on the camera housing. The automated recognition results were highly correlated with the manual counts and they were highly reliable when used to track fish variations at different hourly, daily, and monthly time scales. In addition, our methodology could be easily transferred to other cabled video-observatories.

## Introduction

Recent technological progress has rapidly advanced the exploration of the world’s oceans, opening up new possibilities to address questions related to the variety, distinctiveness and complexity of marine life. Nevertheless, many of the existing technologies still have to be fully transferred to marine sciences. This is particularly needed to develop innovative systems for biological monitoring, to implement them, and to evaluate their performance^1^. Computer vision and machine learning methodologies are expected to offer new tools for use in the marine sciences, and they will have a wide range of applications in ecology and ecosystem management, such as stock assessment and species conservation^2^. As human impacts and global climate change accelerates, one of the most urgent tasks for the coming decades is to develop technologies to continuously track and accurately predict biological responses, which will provide solid guidelines for their management and conservation^3^.

The traditional concerns of marine research and applications have been in quantifying the abundance of species through space and time, and also in understanding patterns of fish behaviour^4^, such as the fisheries management^5^ and aquaculture^6^. Changes in fish communities, especially regarding commercial species, are also considered under relevant international management policy actions, such as in the case of the EC Marine Strategy Framework Directive (2008/56/EC). In this management effort, cameras are increasingly being considered as one of the most promising approaches for biodiversity monitoring^7,8^. Video-information coupled to concomitant environmental monitoring is particularly needed because some species can rapidly shift their distribution according to environmental drivers^9,10^ with complex alterations of the associated ecosystem services^11,12^. With respect to the traditional sampling approaches, video-based fish counts are gaining popularity as an effective, non-invasive sampling method in the marine environment^13^. This technology has been proposed as a valuable and cost-effective complement to expensive *in situ* monitoring programs that are operated through vessels (e.g., trawling and ROV surveys)^14^. Finally, increasing efforts are being made to implement the use of underwater video cameras.

Despite their high deployment and maintenance costs^15^, installing cameras coupled with other biogeochemical and physical sensors allows cabled observatories to provide powerful devices for quantifying biotic components at time frequencies that span from seconds to hours, months, and even years, producing a huge amount of data that urgently needs appropriate methodologies for an effective automated processing^16^. The resulting dataset can be used to establish solid cause-effect relationships between biotic responses (e.g., macro- and megafaunal community changes) and environmental perturbations of either natural or anthropogenic nature. This continuous and coupled observation of biological and environmental parameters represents the core of an ongoing “technological transition”, which will have significant implications for future monitoring strategies^17^.

Computer vision and pattern recognition are key elements of this technological progress and they offer new possibilities to use marine cabled video-platforms. Over the last two decades, a number of methodologies have been proposed for fish species recognition^18^. However, the great variability arising from either divergent species morphologies or from fluctuating conditions in which the videos are captured is still a major challenge for automated processing^4^. Indeed, the methods that have been developed so far have the main shortcoming of having been tested under controlled conditions, such as constrained environments (e.g., fish farms or laboratories) or stable-optimal meteorological conditions (i.e., good water transparency and no or very low turbidity)^19^ or in relation to the classification of single species^20,21^.

Many automated recognition and classification approaches have been experimented and validated on the Fish-4K-knowledge (F4K) repository^22^, which only provides underwater images acquired during the daylight (i.e., from the sunrise to the sunset) in oligotrophic and transparent coral reef waters. These automated approaches span a wide range of topics, from statistics^23^ to convolutional neural networks^13,24,25^ and unsupervised machine learning^26^. These methods are used to recognise, classify, and count fish specimens. Other approaches have been validated through the ROV images that are acquired in the deep-sea light homogeneous environment^27^ or in aquarium trials^28^. In addition, ad-hoc aquaculture devices have been employed to force the fishes to swim frontally to the video cameras^6^.

Methods that are robust enough to handle all of the possible varying conditions of the natural environment are still unavailable^2,4,29^ but are highly requested to transform the cameras on underwater cabled observatories into quantitative sensors for ecological monitoring^30^.

In this study, we have developed a novel video-automated procedure to effectively track and estimate fish count variations, without discriminating among different species, in a challenging real-world scenarios. A general supervised machine learning framework for image content-based fish recognition was conceived and evaluated under different light conditions, variable water turbidity, and changing bio-fouling coverage on the camera housing. We used a K-fold Cross-Validation framework^31^ to select the most relevant image-features^32^ and to produce an automated image classifier with high generalization performance^33^. Tests were performed on more than 20,000 images that were acquired at the OBSEA EMSO testing-site^34^ during the years 2012 and 2013, at 30 min frequency, continuously over day and night. The fish counts and the related time series were validated by comparing automated versus manually generated data.

## Results

**Figure 1:**
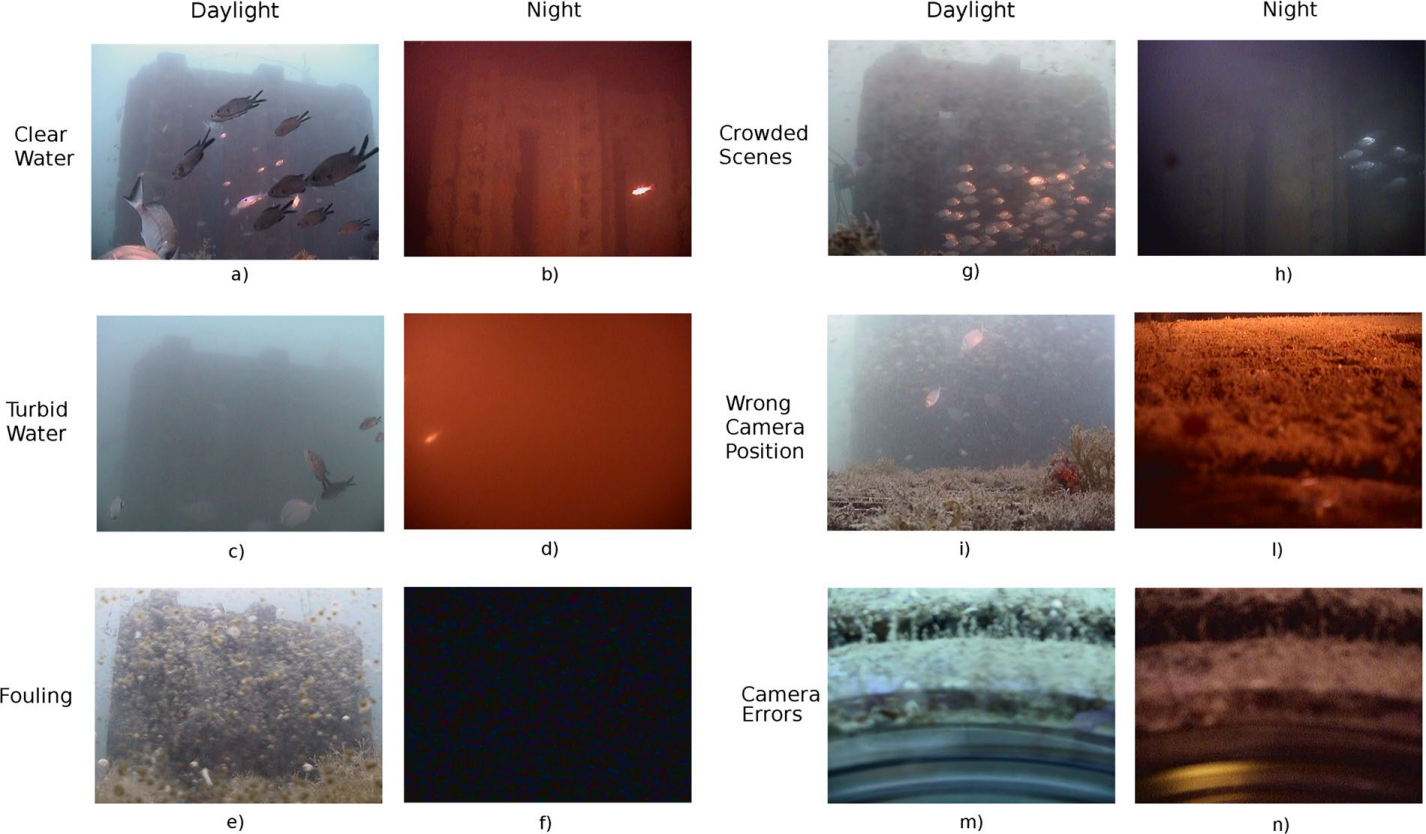
Examples of the most relevant conditions of image acquisition occurring at the OBSEA observatory during the daylight and night: clear and turbid water, bio-fouling on the camera housing, crowded scenes, and wrong pan-tilt-zoom camera positions and errors.

The training and validation dataset that was used for learning the automated image classifier was obtained by randomly sampling a subset of images that were acquired in the year 2012. This dataset covers one year to enable it to capture all of the most critical acquisition conditions that could have affected the quality of the content-based image recognition, including the light variation between day and night, changes in water transparency (i.e., clear vs. turbid water), the bio-fouling on the camera, crowded scenes (i.e., presence of large fish schools), and wrong positions of the Pan-Tilt-Zoom (PTZ) camera; as shown in Fig. 1.

The learnt image classifier was then tested on images that were acquired in the year 2013, where 10,961 images were manually scored according to the degree of water turbidity and bio-fouling present on the camera. This latter score information was used to estimate the effect of both phenomena on the recognition performance.

### Training and Validation of the Image Classifier

Among the images acquired in the year 2012, 11,920 images were available for learning and validating a binary image classifier capable to detect the fishes contained into an image, without discriminating among different species. Given that one of the most critical acquisition conditions is the different light diffusion between daylight (i.e., natural light) and night (i.e., artificial light), the training and validation image dataset was obtained by uniformly sampling 10% of the daylight images and 10% of the night images, which gave a total of 1,191 images corresponding to 10% of all of the images acquired in 2012.

**Figure 2:**
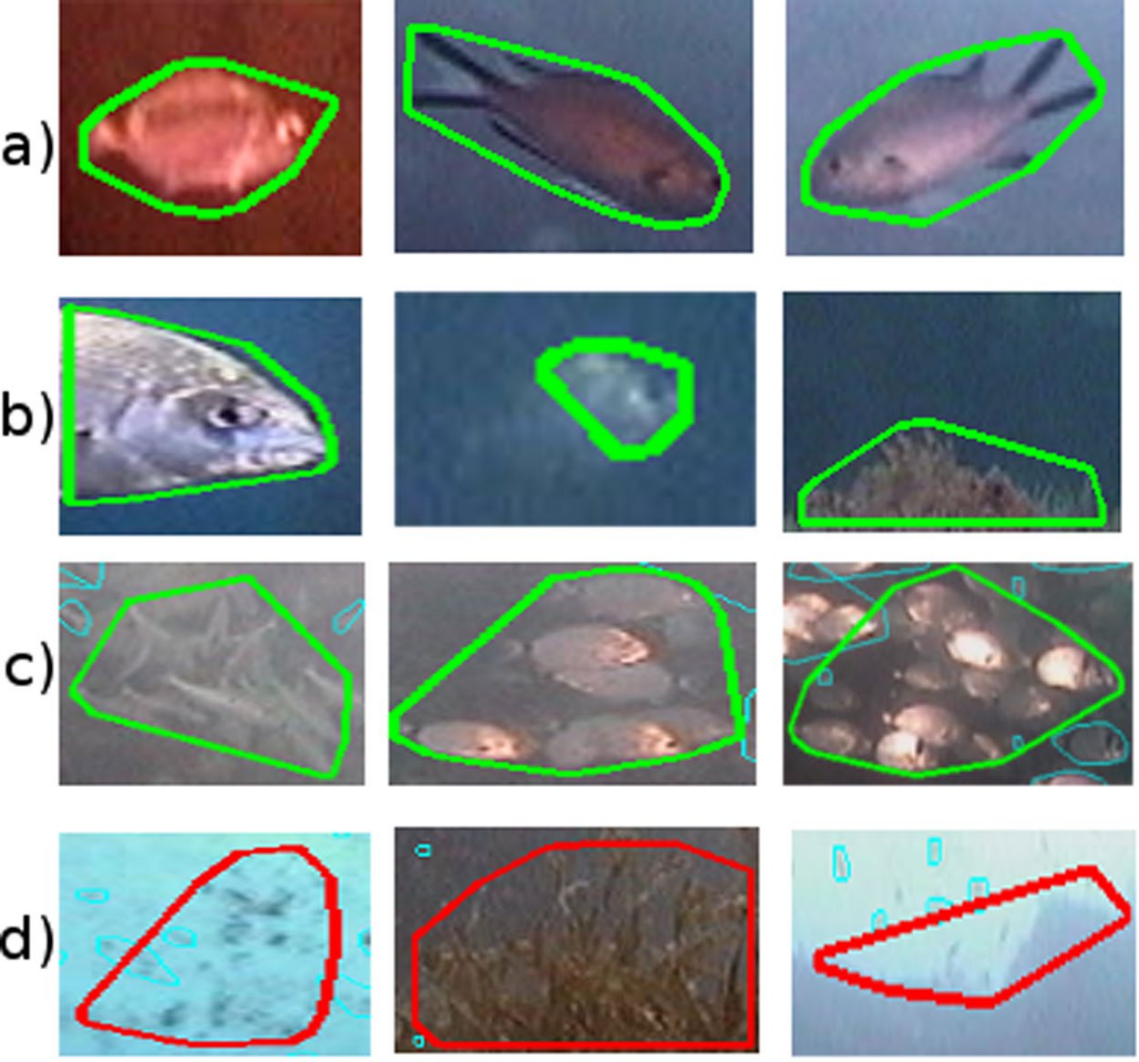
Representative Regions of Interest (RoIs) used in the examples set for training the binary image classifier. The RoIs bounded by a green contour, (**a**), (**b**) and (**c**), correspond to positive examples; while the RoIs bounded by a red contour, (**d**), correspond to negative examples.

The most representative examples of image regions used for training the binary image classifier are shown in Fig. 2. Regions of Interest (RoI) were automatically extracted from the training and validation image dataset and manually labelled to identify the positive and negative examples. Figure 2(a) shows three examples of RoIs labelled as positive examples that contain the whole fish. Figure 2(b) shows three positive examples, although in this case only part of the fish is contained in the RoI. This happened because sometimes the fishes were too close to the border of the image, as shown in the leftmost and in the rightmost images of Fig. 2(b). In other cases, the segmentation process was not able to identify a RoI containing a complete fish (see Fig. 2(b)-middle) because of the position of the fish, the light conditions, and the water turbidity. Large schools of fishes were sometimes captured by the images, as shown in Fig. 2(c). In these cases the fishes were overlapping and, therefore, the segmentation process was not able to produce one RoI for each fish. Although a RoI containing more than one fish can compromise the correct fish count, in the experiments that we performed large school of fishes were split into several RoIs and the magnitude of the fish abundance was still captured. Figure 2(d) shows the three most common situations where the RoIs were labelled as negative examples. In this case, the leftmost image shows a RoI containing some patches of bio-fouling, in the middle image the RoI contains some algae and in the rightmost image the RoI contains the borders of the artificial reef imaged by the camera.

The manual labelling of the RoIs extracted from the image dataset produced 861 positive examples and 27,162 negative examples. After labelling, the image-features were extracted from each RoI. They were then used for the training and the validation of the image classifier within a 10-Fold Cross-Validation framework. To maximize the use of the information contained in the examples set and to perform a balanced cross-validation, the 10-fold cross-validation was performed 10 times, each time randomly sampling 861 negative examples from the examples set and each time, reshuffling the 861 positive examples. The recognition performance obtained from the validation phase resulted in an average accuracy of 92% with a standard deviation (std) equal to 0.02, a true positive rate (TPR) equals 95% with std equals to 0.03 and a false positive rate (FPR) that equals 12% with std equals to 0.04. Table 1 summarises the data that we used for the training of the image classifier and also the corresponding validation performance.

**Table 1:** Summary of the data acquired in the year 2012 that was used to train and validate the binary image classifier.

2012 Image Dataset	Total Acquired Images	11,920
	Daylight images	6,814
	Night images	5,106
Training & Validation based on 10-fold Cross-Validation	Training & Validation Images (10% of the Daylight and Night Images)	1,191
	Positive Examples	861
	Negative Examples	27,162
Validation Performance	Accuracy (std)	92% (0.02)
	True Positive Rate (std)	95% (0.03)
	False Positive Rate (std)	12% (0.04)

### Test of the Image Classifier

The ground-truth that we used in the test phase corresponds to the number of fishes that are visually observed in each of the 10,961 images acquired in the year 2013. The Pearson Correlation between the abundance time series resulting from the observation and the abundance time series produced by the automated image classifier was used to evaluate the test performance.

**Figure 3:**
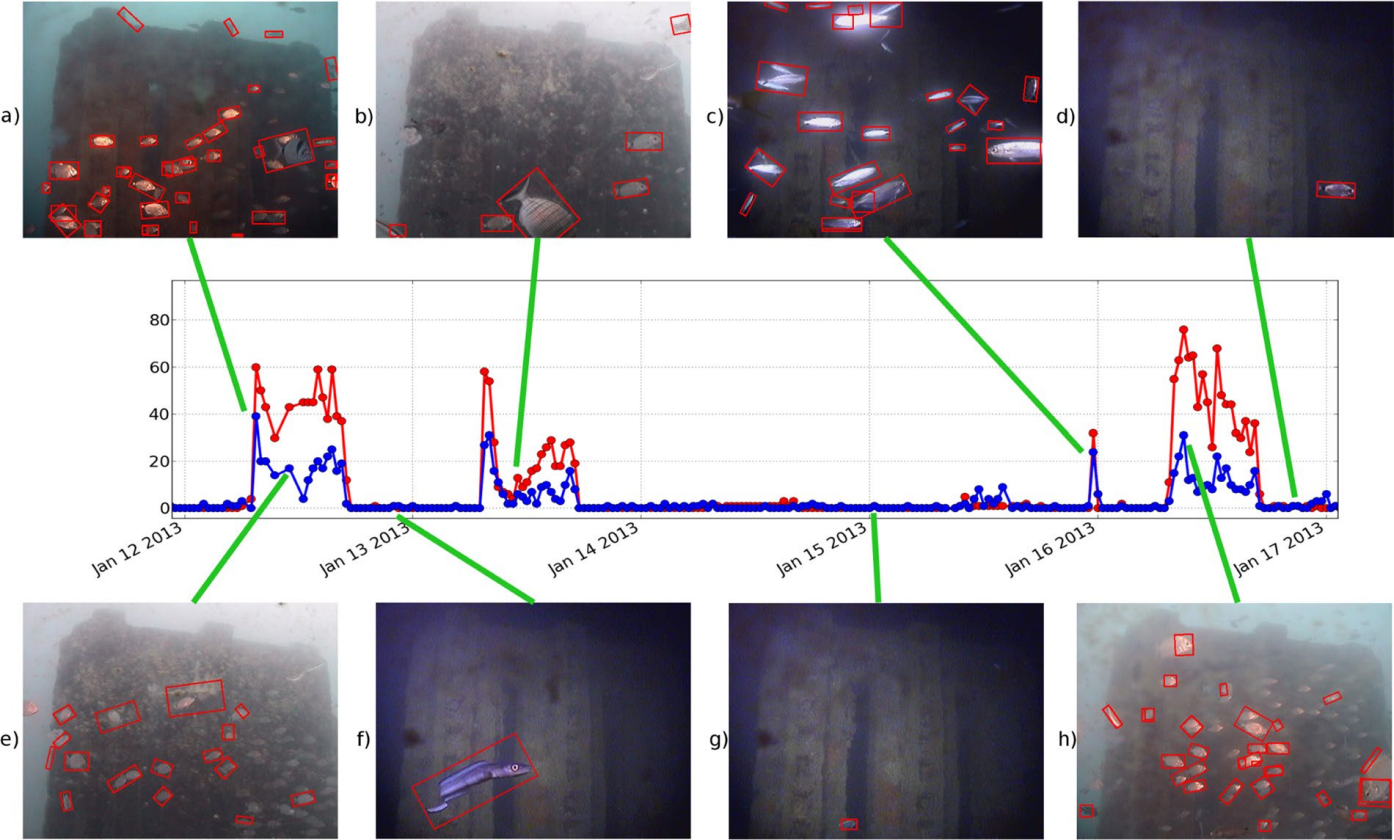
An example of a correlation between the time-series obtained through the visual inspection of the images (red line) and the time-series automatically extracted by the image classifier (blue line). The images show several examples of automated recognition (red boxes) during the presence of moderate turbidity and bio-fouling.

An example of the Pearson correlation between the time series resulting from the observation and the time series produced by the automated image classifier is shown in Fig. 3 (*r* = 0.90, *p* = 1.62^−83^), where a fragment of the time-series obtained through the observation (red line) is compared with the fragment of time-series automatically extracted by the image classifier (blue line) in the same period. Even if the automated recognition underestimated the observed abundance, the temporal variation of the observation is captured by the automated image classifier. In the presence of few observed specimens, few specimens were automatically recognised independently by the light diffusion, the water turbidity and the bio-fouling present on the camera. Analogously, in the presence of observed crowded scenes, many fishes were automatically recognised.

**Figure 4:**
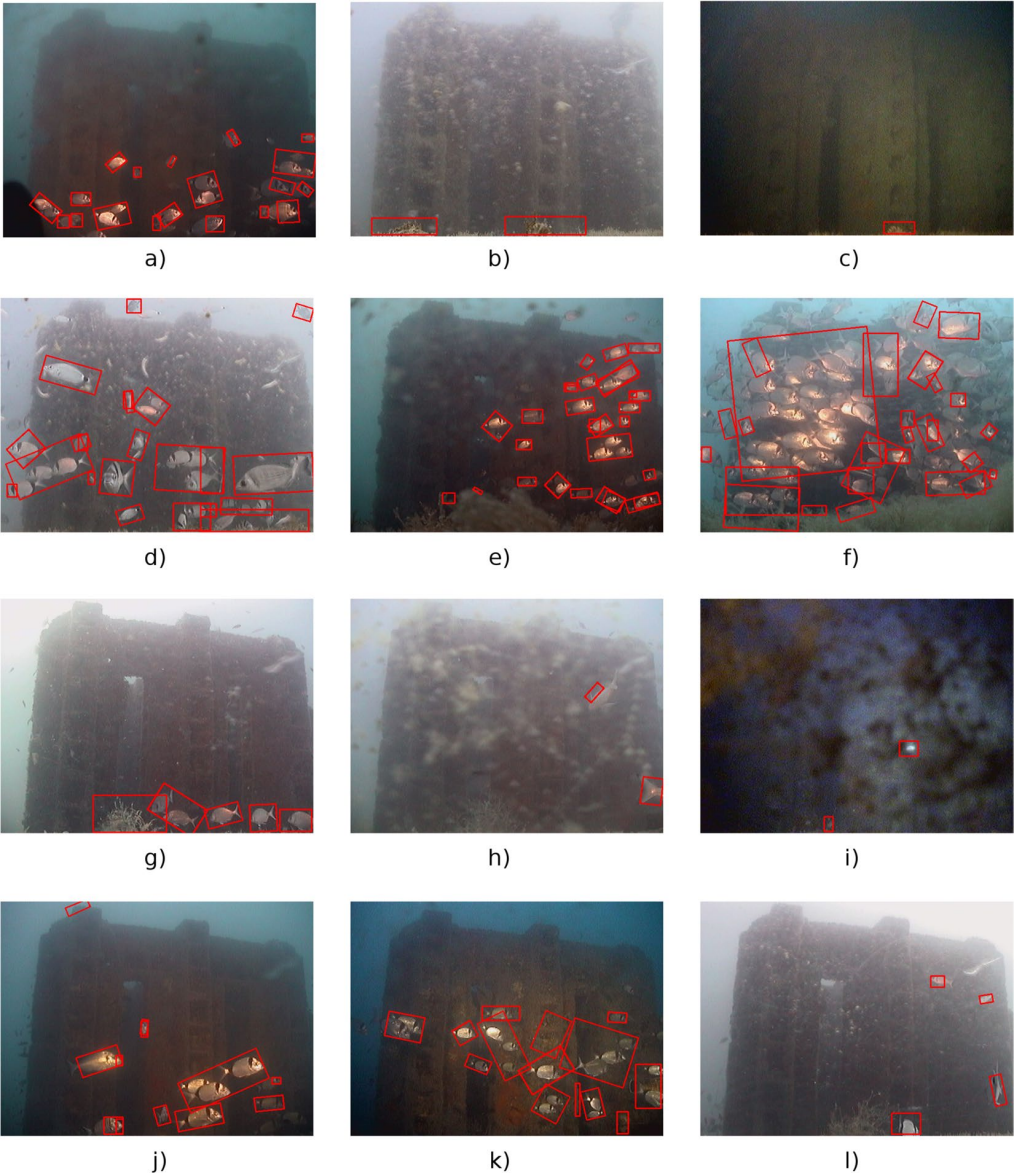
Examples of automatic recognition in different operating conditions: Sunlight with turbid waters (**b**), (**l**); twilight (**c**); presence of mucilage (**e**), (**f**); bio-fouling over the camera (**i**). False negative in correspondence of dense aggregations of fishes are also illustrated (**f**).

The images in Fig. 3(e) and (h) show the recognition results (red boxes) of two crowded scenes acquired during daylight. Similarly, Fig. 3(a) and (c) show the recognition results of similar scenes acquired in the early morning and during the night, respectively. When few specimens were present, the automated image classifier performed a correct recognition, as shown in Fig. 3(b), acquired during the daylight and in Fig. 3(d),(f) and (g), acquired during the night. Figure 4, shows several recognition results sampled from the test dataset. The supplementary video that is provided online shows the automated recognition of a 24 hour time-series fragment.

#### The effects of turbidity and bio-fouling on the automated recognition performance

The correlation between the observed and the automated time series was studied at 30 min. frequency, over daily and monthly scales, and under varying water turbidity and bio-fouling scores. Two different test datasets were used: a complete dataset that included all of the images acquired in the year 2013 and a reduced dataset that was obtained by removing all of the images characterised by the wrong position of the PTZ camera (e.g., Fig. 1(i),(l)), the image errors (e.g., Fig. 1 (m) and(n)) and few very crowded images where a school of fishes takes up the whole scene.

The study of the reduced dataset provided detailed information on how light diffusion, bio-fouling, and water turbidity have affected the automated recognition performance. Similar tests were performed on the complete image dataset, which show how the wrong PTZ positions and the image errors can affect the capability of the image classifier to capture the fish abundance temporal dynamics.

**Figure 5:**
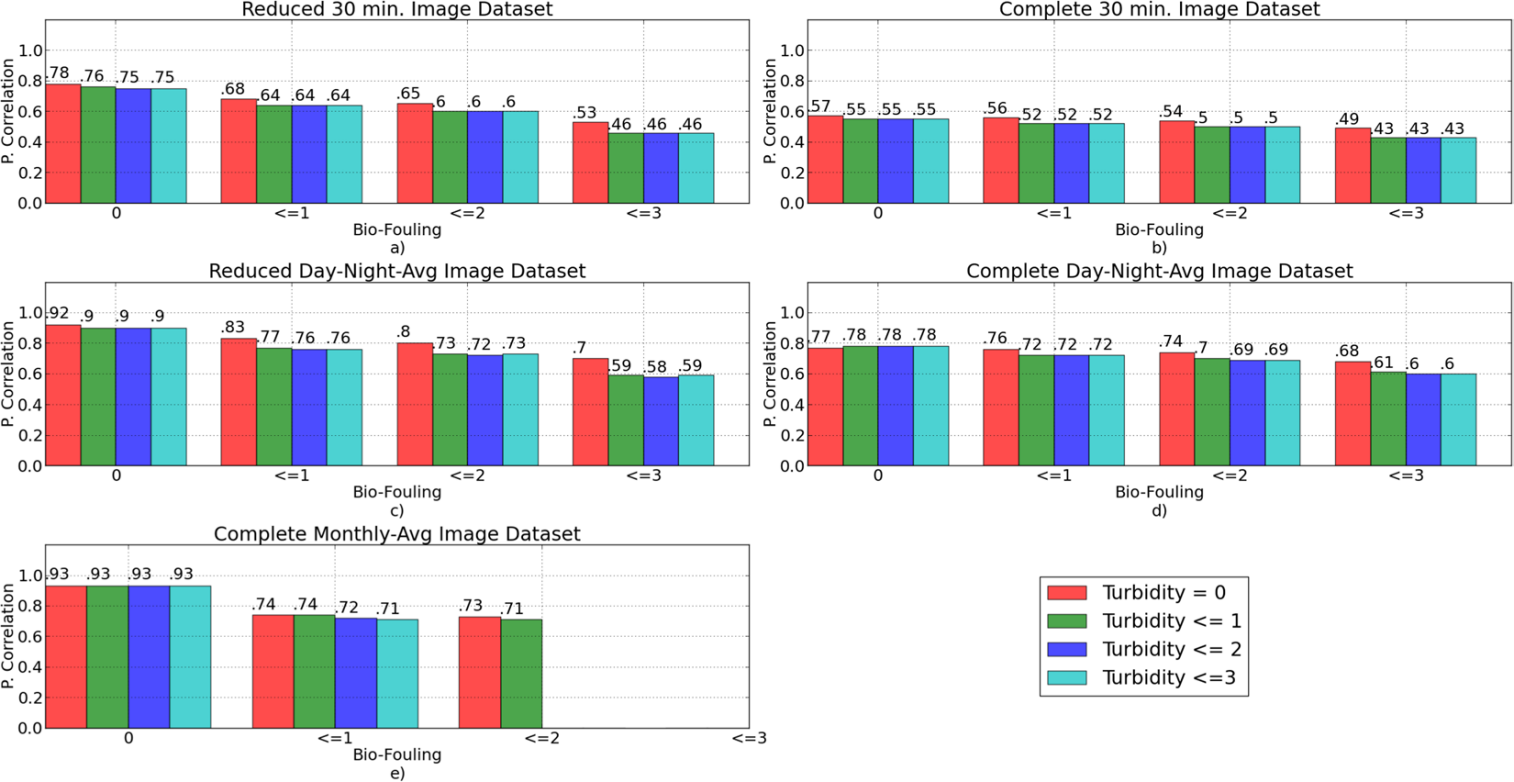
Pearson correlation between the observed and the recognised time series as a function of the level of water turbidity and bio-fouling on the camera housing.

Figure 5 shows the recognition performance at 30 min., daily and monthly scales for both the complete and the reduced datasets in relation to variable bio-fouling and turbidity. Globally, as the bio-fouling score increases (from 0 to 3), the correlation between both automated and manual time series decreases. This reveals a sensible decline in automated classification performance. In contrast, the level of water turbidity does not relevantly affect the correlation between the observed and the recognised time series.

Considering the reduced 30 min. image dataset shown in Fig. 5(a), with the absence of, or with a small quantity of bio-fouling (i.e., bio-fouling score equal to 0), the correlation between the two time-series shows just a small variation from 0.78 to 0.75 (*p* ≤ 0.001), even if the water turbidity score is increased. Moreover, independent of the water turbidity level, the correlation between the observed and the recognised time-series decreases below 0.60 only in the case of a heavy presence of bio-fouling on the camera (i.e., bio-fouling score equal to 3 is considered).

Figure 5(b) shows the results obtained by considering the complete image dataset. Similar to the previous case, the water turbidity intensity does not affect the recognition performance. In contrast, as the bio-fouling score increases, the correlation between both manual and automated time series decreases. Therefore, the recognition performance is sensibly reduced, even if the level of bio-fouling is low (i.e., score equal to 0). This shows the bad effects of PTZ and image errors. In this case, the correlation between the observed and the automate time-series is 0.57 and it decreases to 0.43 as the bio-fouling level increases (*p* ≤ 0.001).

The daily dynamics of the fish abundance were analysed by averaging the number of observed and recognised fishes in the images acquired during daylight and during the night. The daylight and the night periods were computed according to the global solar irradiance data that were provided by the sensors installed on the OBSEA. Similar to the 30 min. dynamics, as the bio-fouling increases, the correlation between the observed and the recognised time-series decreases; as shown in Fig. 5 (c) and(d). When the PTZ positions were wrong and the image errors are not considered in the analysed dataset (Fig. 5c) the automated recognition produces a time-series that is strongly correlated to the manual inspection, even if the presence of bio-fouling on the camera is heavy and independent of the water turbidity. In fact, the correlation is still 0.7 (*p* ≤ 0.001) when images with bio-fouling and turbidity scores equal to 3 are considered.

Similar to the previous cases, the wrong PTZ position of the camera and the image errors sensibly decrease the recognition performance; as shown in Fig. 5(d). Nevertheless, by considering the complete Day–Night averaged dataset, the correlation between both automated and manual time series varies from a maximum of 0.78 (*p* ≤ 0.001) when the bio-fouling score is 0 to a minimum of 0.60 (*p* ≤ 0.001) when images with bio-fouling score equal to 3 are considered.

Finally, the monthly dynamics of the fish abundance were studied by computing the monthly average of the fish specimens number observed and recognised in the two image datasets. As shown in Fig. 5(e), because few elements were used to compute the correlation between both series, the automated recognition of images with the heavy presence of bio-fouling (scores = 3) could not be correlated to the results of the visual inspection (*p* ≥ 0.001). Similar results were obtained for the images characterised by moderate or heavy bio-fouling and moderate or high turbidity, and for the reduced monthly average dataset. Nevertheless, as for the previous analysis, low levels of bio-fouling resulted in a strong correlation between both time series, independent of the water turbidity level.

### Efficacy of the Automated Recognition for Ecological Analyses

Our results showed that bio-fouling affects the results obtained by using recognized and observed data more than turbidity. Globally, significant differences between daylight and night data were always detected at all combinations of turbidity and fouling, with decreasing F values (Univariate PERMANOVA test), but still significant at a greater level of fouling (score equal to 3); as shown in Table 2.

**Table 2:** Results of Univariate PERMANOVA test carried out on factor “day vs. night”, comparing observed and recognised datasets with different combinations of fouling (F) and turbidity (T). Both parameters varied from 0 (no or scarce value of the parameter) to 3 (maximum value). ***p < 0.001.

	Observed	Recognised
T0, F0	68.25***	67.83***
T1, F0	53.10***	32.21***
T3, F0	21.87***	15.43***
T0, F1	72.54***	69.10***
T0, F3	121.49***	122.42***
T3, F3	35.45***	32.29***

Turbidity had less affect on data comparisons, with a good correspondence in the results of PERMANOVA at all levels of turbidity (from T0 to T3) but with the absence or small quantity of fouling (F0). Although the PERMANOVA test was always significant, the source of variation (i.e., differences between contiguous months) changed according to the combination of turbidity and fouling conditions. In fact, when the bio-fouling score was equal to 0, PERMANOVA showed a good correspondence in changes of fish abundance between months, when comparing observed and recognized datasets; as shown in Table 3. Conversely, by increasing the bio-fouling score, the correspondence was null; as shown in the Supplementary Table S6.

**Table 3:** Main PERMANOVA test and pairwise comparisons (only significant variations are shown) of observed and recognized abundance data by month, regarding absent or small quantity of bio-fouling (F = 0) and different combinations of turbidity score (T). Numbers from 1 to 12 in the pairwise tests indicate months from January (1) to December (12).

	observed		recognised	
	main test			
T0, F0	Pseudo-*F*_9,64_ = 5.21***		Pseudo-*F*_9,64_ = 3.76**	
	**pairwise**			
	**Groups**	**t**	**Groups**	**t**
	1, 2	2.96*	1, 2	2.37*
	4, 5	2.32*	4, 5	2.38*
	8, 9	3.93**	8, 9	2.35q.s
				q.s = 0.050
T1, F0	Pseudo-*F*_9,73_ = 6.15***		Pseudo-*F*_9,73_ = 2.37*	
	**pairwise**			
	**Groups**	**t**	**Groups**	**t**
	1,2	3.44**	1,2	2.36*
	4,5	2.14*	4,5	2.17*
	8,9	3.85**		
T3, F0	Pseudo-*F*_9,64_ = 5.99***		Pseudo-*F*_9,74_ = 3.15**	
	**pairwise**			
	**Groups**	**t**	**Groups**	**t**
	1, 2	3.81**	1, 2	2.75*
	8, 9	2.87*		

Finally, GLM models related the abundance data with the environmental variables, which provided similar results: the driving variables of changes in fish abundance were similar when running the models with observed and recognized datasets with absent or small quantity of fouling (F0) and at all levels of turbidity; as shown in Table 4. Meanwhile, different variables drove changes in the observed and recognised datasets if fouling was set at level 1 or 3; see the Supplementary Table S7.

**Table 4:** Results of GLM models for absent or small quantity of bio-fouling (F = 0), and different values of water turbidity (T). Chla_1mo = Chlorophyll-a concentration recorded by satellite one month before actual data; SST_sat = sea surface temperature recorded by satellite; solar_irr = solar irradiance; corr = sign of the correlation; neg = negative.

	Observed							Recognised						
	Vars	Df	Dev.	Res. Df	Res. dev.	F	sign corr.	Vars	Df	Dev.	Res. Df	Res. dev.	F	sign corr.
**T0-F0**	NULL			64	14.44			NULL			64	6.8771		
	Chla_1mo	1	1.12	63	13.317	5.31*	neg	Chlor_1mo	1	1.09	63	5.7839	11.91**	neg
	AIC = 87.41	expl. Dev. = 7.78%						AIC = 33.21	expl. Dev. = 15.90%					
**T1-F0**	NULL			73	231.82			NULL			73	79.88		
	Solar irr	1	19.75	72	212.07	6.70*	neg	Solar_irr	1	4.54	72	75.34	4.53*	neg
								Chla_1mo	1	4.37	71	70.97	4.37*	neg
	AIC = 293.91	expl. Devi. = 8.52%						AIC = 214.91	expl. Dev. = 11.15%					
**T3-F0**	NULL			72	190			NULL			72	65.35		
	Solar_irr	1	39.14	71	150.86	18.42***	neg	Solar_irr	1	11.18	71	59.93	14.11***	neg
								Chla_sim	1	4.46	70	55.47	5.62*	neg
	AIC = 266.15	expl. Dev. = 20.6%						AIC = 195.12	expl. dev. = 21.99%					

## Discussion

In this study, we developed a novel methodology for automated fish recognition and counting at a cabled video-observatory, which allowed us to take into account a variety of operating circumstances that included wide variations in light intensity, turbidity, fouling growth and dense fish assemblages. This technique was tested at a high frequency (i.e., 30 min) and over a long–lasting period of time (i.e., one year). We found that it is a reliable benchmark for scaling to other still video sources, either in deeper continental margin areas–such as in the LoVE observatory^35^ and the NEPTUNE network^36^)–or on board of mobile platforms–such as ARGO floats, ROVs, AUVs^33,37,38^ and crawlers^39,40^ if the hypothesis that the not relevant information (e.g. background, bio-fouling over the camera) changes more slowly, along the time, than the relevant subjects (e.g. the fishes) contained in the acquired images. This condition is satisfied especially when the background is fixed or it consists only of water column or it is mostly uniform (e.g. a sandy seabed).

The light diffusion followed transient changes in cloud coverage. It varied daily, from the dawn to the sunset, and it varied seasonally (due to the changing photophase length). During the night, the use of artificial illumination imposed entirely new conditions, where the intensity generally diffused with a more homogeneous field than daytime. This also affected the scattering effects of the suspended particulate and the fish bodies^41,42^. All of these luminosity changes can severely affect the fish recognition performance; for example, by changing the animal textural features or their contrast with the background. Nevertheless, our method proved to be robust enough to handle these changes. It was also robust when the artificial light changed both the background and the foreground subjects, making some fish almost invisible and other fish strongly highlighted thanks to the light reflections on their skin markings.

Unexpected variations of the water turbidity occurred in relatively short times (e.g., hours) and they persisted for several days, as in many other coastal areas^43^. Turbid waters and changes in light diffusion reduced the camera’s field of view. This challenged the recognition of fish distant from the camera, which became similar to patches of bio-fouling. In this case, not all of the fish in the scene could be correctly detected, which increased the false negative rate. The method was, however, capable of handling water turbidity (see Fig. 5).

Fouling organisms typically grow over any solid exposed surface. This is a typical issue for the use of stationary underwater cameras, although solutions such as protective coating, wipers, and copper shutters are available^44–46^. In our case, fouling was generally present and was subjected to seasonal variations because these communities grow less during the cold periods and flourish during the warm season^47^. As expected, as the bio-fouling developed on the camera porthole and on the lighting system, it had more affect on the recognition performance; for example, it reduced the Pearson correlation between the observed and the recognised time series. While absent or moderate levels of bio-fouling can be effectively managed by our automated classifier independent of the light diffusion and of the level of water turbidity, larger patches tend to occlude the scene and these corrupt the recognition accuracy.

Another constraint limiting recognition efficiency was represented by “crowded scenes”, when large numbers of fish gather together in front of the camera. When these assemblages are particularly dense, individuals typically overlap each other. This increases the false negative rate. Although these situations may be critical for automated counting, our results demonstrated that in presence of low or moderate levels of bio-fouling, the correlation between observed and the automated time-series is still high; it is even high when dense schools of fish gather.

The ultimate aim of our automation process is to use it to study natural processes. Consequently, it is important to be able to efficiently correlate the seasonal abundance changes with other biotic and environmental factors by using consistent manually counted and automated recognised time series. Indeed, the two datasets provided highly comparable results at all levels of light diffusion and turbidity. Accordingly, the present imaging processing scripts represent a contribution towards promoting the use of cameras as autonomous sensors for the quantification of ecosystem functioning in different marine ecosystems based on faunal quantification^17^.

The results show that the recognition performance is compromised when the Pan-Tilt-Zoom camera installed on the observatory assumes unexpected positions or when an image transmission error occurs. These acquisition conditions occurred a smaller number of times than the light, water, and fouling condition changes. Consequently, they are not sufficiently represented in the set of examples used within the proposed supervised machine learning approach. Therefore, the learnt automated classifier was not able to manage these images and the number of false positive detections increased. This issue can be consistently attenuated by building an ad-hoc set of examples with the aim of managing the camera malfunctions. Nevertheless, because the camera malfunctions should be avoided independent of the image analysis activities, no actions were taken in this work for adapting the training set.

The results show that the recognition performance is mostly affected by the bio-fouling and by the system errors. In fact, the light radiation changes and the fish crowding are ubiquitous in the image dataset and their combined effects on the recognition performance is marginal and can be ignored.

The proposed image analysis and recognition approach was conceived to adaptively work in different environmental and operational contexts (i.e., at all depths of the continental margin, over heterogeneous and homogeneous backgrounds, and with fixed and mobile platforms). Different image segmentation and pattern recognition approaches can be considered, mainly depending on the specific acquisition conditions and on the specific hardware support that executes the software components. If the automated recognition is operated by a CPU with low computational power (e.g., mobile platforms or fixed platforms powered by batteries), the computational complexity of the software components must be limited^33,37^. In contrast, if the automated recognition is not subjected to such limits (e.g., cabled observatories), then different image segmentation or feature extraction approaches can be used^48,49^. Alternatively, the image enhancement and the image differencing methodologies proposed in this work can easily be combined with traditional and novel deep learning approaches^50–52^. Our results were obtained through an easily customisable image elaboration process and pattern recognition approach. We were able to show that the automated extraction of time-series can be embedded and then performed on hardware supported with low computational performance. Moreover, the general character of the proposed methodology is also guaranteed by the flexibility of the supervised machine learning approach. In fact, this methodology can be used for different image backgrounds (e.g., water column, seabed, artificial reefs, shallow or deep water) or for different organisms (e.g., gelatinous zooplankton or fishes)^33^.

The proposed binary classifier for image recognition can easily be extended to multi-classification applications. For example, different binary classifiers can be trained to recognise relevant subjects (e.g., different fish species) and then combined into an ensemble to obtain a multi-classification of all of the relevant subjects contained in the input image^53,54^. In this case, a multi-species time-series that is obtained by using an ensemble of binary classifiers can be used to investigate species assemblage dynamics or species behaviour in a monitored area.

If we consider that these cameras are now being permanently installed worldwide, their potential to track spatio-temporal changes in marine populations and the impacts on ecosystem services is enormous^17^. Nevertheless, the full potential of this technology will only be expressed through the application of automated routines for video-counting. This study has tackled the overall challenge of counting fish in uncontrolled environments and it has provided a robust tool for automated fish counts across multiple depths and habitats. Future research could start transferring and standardising these automated techniques to other existing cabled observatories, which will help to coordinate monitoring efforts toward a synchronous and promising large-scale continuous monitoring of marine ecosystems.

## Methods

The image analysis and recognition methodology proposed in this work combines an image segmentation process and an image-feature extraction process, together with a supervised machine learning approach. This is coupled with a K-fold cross-validation framework that is similar to the approach proposed in^33^. Details on the proposed methodology can be found in the Supplementary Table S1 that is available online.

### The Image Dataset

The content-based image recognition algorithm for fish counting was learnt and tested by using the image dataset provided by the Western Mediterranean Expandable SEAfloor OBservatory (OBSEA)^34^ (the data can be requested through the contact section of the website) in the years 2012 and 2013. The OBSEA is located at a depth of 20 m within the Colls i Miralpeix Marine Reserve, which is 4 km off Vilanova i la GeltrÃ° (Catalonia, Spain)^55^. This is the location of a testing site for the European Multidisciplinary Seafloor and water-column Observatory (EMSO). This location is equipped with an OPT-06 Underwater IP Camera (OpticCam) associated to two LED-based lighting sources located beside the camera at 1 m distance from each other, emitting 2900 lumen with colour temperature of 2700 kelvin and with an illumination angle of 120°^55^. The camera acquires digital images of the surrounding environment at 360° at 30 min frequency, continuously by day and night.

### The Environmental Data

The GLM analysis was based on several oceanographic and atmospheric parameters: the water temperature, the pressure of the water column, the water salinity, the air temperature, the wind speed and direction, the global solar irradiance, the sun elevation and azimuth can be accessed through the web portal of the OBSEA observatory^34^; while the chlorophyll a concentration recorded from three to one month before and simultaneously to actual data, was downloaded by the NASA hearth data science portal Giovanni^56^.

### Image Segmentation and Feature Extraction

The proposed segmentation process is aimed at reducing the effects of the light diffusion changes, the effects of the turbid water and the effects of the bio-fouling presence on the camera. This process is based on two assumptions: (i) the images are sorted with respect to the acquisition time (i.e., the image dataset must be organised as time-series), and (ii) considering the time-sorted sequence of the images, the relevant subjects within an image (i.e., fishes) change faster (along the sequence) than the not relevant image regions (e.g., background, bio-fouling). Within these two assumptions, an image differencing approach between consecutive images^57^ was used to discard image regions persisting along consecutive images (i.e., representing not relevant subjects, such as image background) and at the same time preserve image regions containing differences between consecutive images (i.e., representing candidate relevant subjects, such as fishes). An example of image differencing is shown in the Supplementary Fig. S2, where fishes are even detected in the presence of massive bio-fouling on the camera.

After the image difference is computed, a blurring operator, a Gaussian thresholding and morphological operators^57,58^ were used to identify the image blobs representing potential relevant subjects (i.e., fish bodies) and the algorithm presented in^59^ was used to extract the corresponding blob contours. Due to the natural propensity of fishes to hide so that they can prey without being predated, the light diffusion (either natural or artificial) on the body surface and its orientation with respect to the camera often produce blobs that are characterised by jagged contours that do not correspond to the proper fish silhouette. This misleading effect was reduced by characterising the blob contour through its convex hull.

The convex hulls identified on the image difference were then mapped back onto the original image. The RoIs corresponding to the bounding boxes of these convex hulls were then analysed to extract the image features that are able to describe both the texture and the shape of the corresponding potential relevant subjects^48,57,60^. The image features used in this work are detailed in the Supplementary Tables S1 and in S2.

### Image Recognition and Feature Selection

The recognition problem faced in this work corresponds to the detection of one or more fishes within each analysed image. To achieve this task, a binary classifier is defined on the RoIs extracted from the input images, where the returned output assumes a value 1 if the RoI contains at least a fish and is 0 otherwise.

The binary classifier is learnt through a supervised machine learning approach that combines a genetic programming (GP) based procedure with a stratified K-fold cross-validation framework; as discussed in^33^. The cross validation framework allows to select the most relevant and effective image features^32,33^ and at the same time assures good generalization performance of the binary RoI classifier. The GP parameters that were used to learn the automated image recognition algorithm are shown in the Supplementary Table S3.

Within the supervised learning phase, a 10-fold cross-validation was used to train and validate the binary classifier, where the positive and negative examples were obtained by manually labelling the RoIs extracted from the 10% of the images acquired in the year 2012. The image features resulting from the training and the validation process are shown in the Supplementary Table S4 online, while the automated image classifier is defined by the Supplementary equation Eq. S1 online, based on the GP individuals shown in the Supplementary Table S5.

The validation performance of the learnt classifier was then obtained by computing the average and standard deviation of accuracy (ACC), true positive rate (TPR) and false positive rate (FPR), which are defined as:

$$ACC=\frac{TP+TN}{TP+FP+FN+TN},$$

$$TPR=\frac{TP}{TP+FN},$$

$$FPR=\frac{FP}{FP+TN},$$

where *TP*, *FP*, *TN* and *FN* represent true positive, false positive, true negative and false negative recognitions, respectively; as discussed in^61^.

The ground-truth that was used in the test phase of the binary classifier corresponds to the number of fishes per image of the test dataset, which were obtained through visual counting performed by expert biologists. In this case, the effectiveness of the binary classifier was estimated by computing the Pearson correlation between the time series resulting from the visual inspection and the time series produced by the automated image classifier. The correlation between the two time-series indicates the ability of the automate image classifier to capture the same temporal dynamics that were identified through the visual inspection.

Besides the visual counting, the images collected in the year 2013 were also manually tagged to represent the level of water turbidity and bio-fouling on the camera. These images were used in the test phase to estimate the impact of this of phenomena on the quality of the automated recognition. The scores that we used to describe the level of water turbidity varies from 0 to 3, as follows: clear water, low turbidity, moderate turbidity and high turbidity, respectively. Analogously, the scores that we used to describe the amount of bio-fouling also varies between 0 and 3, as follows: absence or a small quantity of bio-fouling on the camera, low level of bio-fouling, moderate bio-fouling and heavy presence of bio-fouling on the camera, respectively.

### Ecological Statistical Analysis

Different statistical analyses were carried out to assess the effectiveness of the automated recognition process in terms of ecological monitoring applications, the inputs were manually and automated fish count data.

For all of the analyses, the data were averaged each 24-h, but considering day versus night samples separately. First, an univariate PERMANOVA^62^ was run on the Euclidean resemblance matrix of square root-transformed abundance data to test for differences between day versus night abundances. Since the univariate PERMANOVA proved that there were significant differences, the subsequent analyses were only focused on the daytime data because the fish abundance at night was considerably lower. Univariate PERMANOVA^62^ was also performed on the “month” factor to check for seasonal temporal differences in automation versus manual counting. Pairwise comparisons were also carried out to assess their level of significance.

Finally, daytime count data were compared with environmental variables using generalized linear models (GLM), where the distribution family used was Gaussian. The model selection was based on minimising Akaike’s information criterion (AIC) values. Before the analysis, a Draftsman plot was performed on the environmental dataset to look for auto-correlation among the variables. Only data that were not auto-correlated (Pearson’s correlation, *R* < 0.70) were retained for the analysis.

The results were compared to assess which level of turbidity and fouling prevented the use of recognised datasets for ecological analyses. Six different datasets were analysed: three different combinations of fouling intensity (F) as 0, 1, and 3 in no turbidity (T) condition (T0-F0, T0-F1 and T0-F3, respectively); and two antithetic conditions of turbidity (0 and 3) with two different conditions of fouling, from no to a maximum in fouling (T1-F0, T3-F0 and T3-F3, respectively).

## Electronic supplementary material

Automated recognition 24hours movie

Supplementary Material
