# Supplementary material for: Tracking Fish Abundance by Underwater Image Recognition
Source: Sci Rep. 2018 Sep 13;8:13748. doi: 10.1038/s41598-018-32089-8 (PMC6137190; doi:10.1038/s41598-018-32089-8)
Supplement: Supplementary file 2 — Supplementary Material [file 41598_2018_32089_MOESM2_ESM.pdf]

Supplementary Information for the paper:

Tracking Fish Abundance by Underwater Image Recognition

Simone Marini*[1] , Emanuela Fanelli[2], Valerio Sbragaglia[3], Ernesto Azzurro[4,5], Joquin Del Rio Fernandez[5], Jacopo Aguzzi[6]

[1] National Research Council of Italy, Institute of Marine Sciences, Forte Santa Teresa, 19132 La Spezia, Italy

[2] Department of Life and Environmental Sciences, Polytechnic University of Marche, Via Brecce Bianche, Ancona, Italy

[3] Department of Biology and Ecology of Fishes, Leibniz-Institute of Freshwater Ecology and Inland Fisheries, Müggelseedamm 310, Berlin, 12587, Germany

[4] Institute for Environmental Protection and Research (ISPRA), Livorno, Italy

[5] Zoological Station A. Dohrn, Villa comunale, Napoli, Italy

[6] Sarti Research Group. Electronics Deparment. Universitat Politecnica de Catalunya (UPC) - Rambla de l'exposició 24, 08800, Vilanova i la Geltrú, Spain

[7] Instituto de Ciencias del Mar (ICM-CSIC). Paseo Martimo de la Barceloneta, 37-49. 08003, Barcelona, Spain

* Corresponding Author: simone.marini@sp.ismar.cnr.it

1 Image segmentation and feature extraction

Figure S1 summarizes the image elaboration tasks for learning and for executing the automated image recognition algorithm. All the image elaboration tasks shown in the two pipelines were implemented in Python, using the OpenCV library [1].

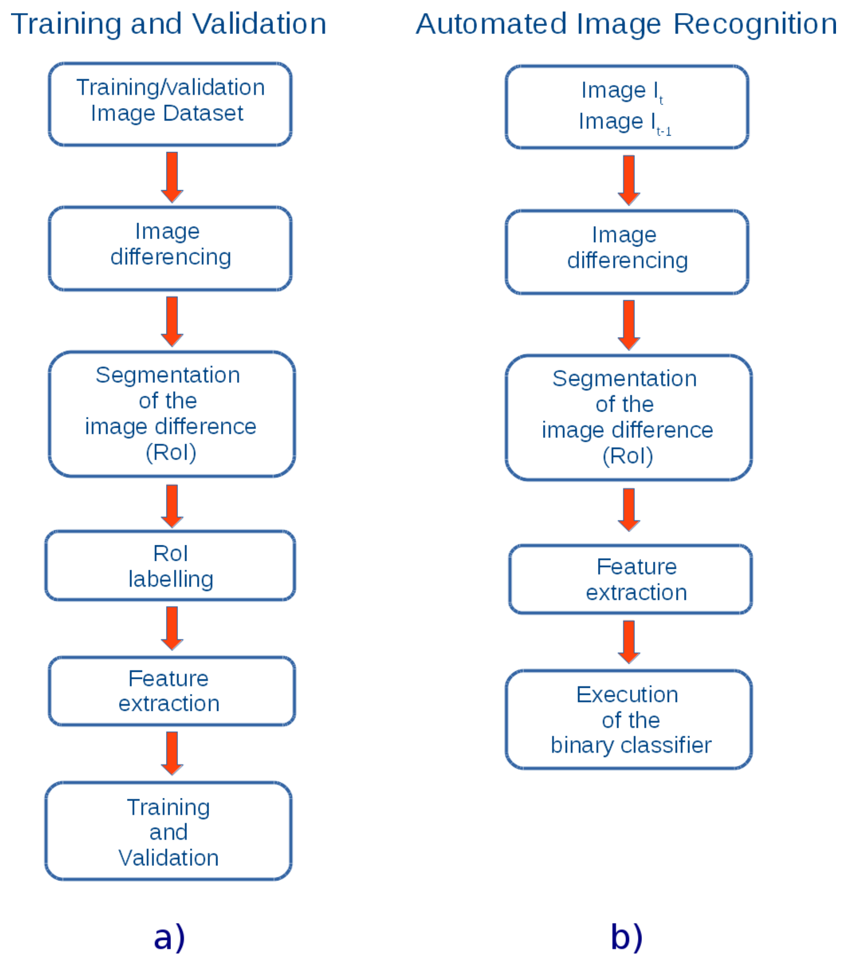

S 1: Schematic representation of the pipelines used for learning the image binary classifier a), and for executing the automated image recognition b).

1.1 Training and validation pipeline

The training and validation phase is aimed at learning the binary classifier capable to automatically recognise the content of an image region following the methodology discussed in [4].

The proposed approach learns the binary classifier from a set of positive and negative examples, whithin a supervised machine learning approach [3, 5]. The example set is obtained through the sequence of tasks shown in the Figure S1a) and described below.

Image Differencing: the image dataset used for the training and validation is organised as a time-series where each image is characterised by a time stamp. This time organisation allows the use of the image differencing approach discussed in [8]. The image differencing removes the image regions that do not changes between consecutive images, as for example the background and the patch of bio-fouling on the camera port-hole. At the same time the image differencing highlights the image regions that changes along consecutive images (e.g. fish samples). An example of image differencing between two consecutive images acquired at the time t and the time $t-1$ is shown in Figure S2. Other techniques for image differencing involving images acquired at the time t, time $t-1$ and time $t+1$ can also be implemented with the same objective and are also discussed in [8].

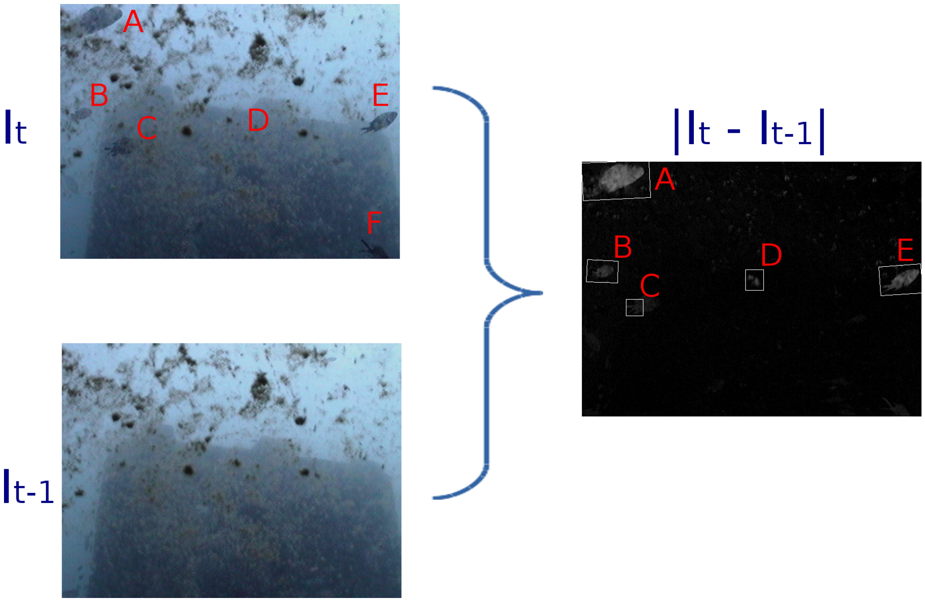

S 2: An exmaple of image differencing [8] between two consecutive images acquired at time t and at time *t-1*. Letters A, B, C, D and E in the image $|I_t - I_{t-1}|$ represent the computed differences between the images I_t and I_{t-1}. In particular, the regions A, B, C, E and F in figure I_t contain fishes, while the region D contains a patch of bio-fouling. Due to the very low contrast in the lower right corner of the images I_t and I_{t-1}, the fish specimen F do not emerge in the image difference.

Image Segmentation: the obtained image difference is then segmented in order to obtain Regions of Interest (RoI) potentially containing fish specimens. Firstly the image difference is blurred with a bi-lateral filter with the aim of removing possible noise generated by the differencing task, while keeping sharp the edges of foreground subjects. Then a Gaussian adaptive thresholding and a morphological opening operator are applied in order to binarise the difference image and remove small not relevant image blobs [8, 1]. For each binary blob identified on the image difference, the algorithm defined in [2, 1] was used for extracting the region contour. The convex hull of each blob is then computed and mapped on the input image acquired at time t.

Region Labelling: the RoI identified on the input image is then labelled in order to define the set of positive and negative examples used for learning the RoI binary classifier. In particular each RoI identified by the segmentation task is visually inspected and manually labelled with 1 if it contains at least a fish specimen and labelled with 0 otherwise. A software component with a simple user interface was defined for performing the manual RoI labelling.

Feature Extraction: the bounding box of each labelled RoI was computed and the image features representing the bounding box interior was extracted according to the tables S1 and S2. Within the geometric image features shown in Table S1, the length of the minor and major axis (*axm*, *axM*), the convex hull perimeter (*perimeter*), the convex hull and the bounding box areas (*cntArea*, *bbArea*) are all expressed as number of pixels and describe the size of the relevant subject. The eccentricity (*ecc*), the equivalent diameter (*equiDiameter*) and the aspect ratio (*aspectRatio*) of the convex hull, together with the extent (*extent*) and the solidity (*solidity*) of the RoI content describe the shape of the relevant subject.

Within the texture image features shown in Table S2, the histogram shape index (*histIndex*) captures the overall pixel intensity variance inside the analysed region. It is obtained by transforming the region into a grey level image and extracting the histogram h of the pixel intensities. Similarly the standard deviation of the mean grey level (*std*) captures the variation of the pixel intensity with respect to the region mean grey intensity μ. The entropy (*ent*) of h captures the information stored in the region, and finally, the normalized contrast index (*contrast*) is defined as the ratio between the difference in the mean grey level inside the region ($mean(gInt)$) and outside the region, but within the oriented bounding box ($mean(gExt)$), and the mean grey level inside the whole bounding box.

All the previously discussed image features were chosen such that they have a linear computational cost with respect to the number of pixels. Moreover, though a single image feature can appear not relevant with respect to the recognition of fish specimens, it can become relevant if combined with other image features.

Table S 1: The geometric image features extracted from each single Region of Interest (RoI).

	feature name	feature meaning	feature computation
**geometric features**	*axm*	bounding box minor axis	
	axM	bounding box major axis	
	perimeter	convex hull perimeter	
	cntArea	convex hull area	
	bbArea	bounding box area	$axM \cdot axm$
	ecc	eccentricity	$\frac{\sqrt{axM^2-axm^2}}{axM}$
	equiDiameter	equivalent diameter	$\sqrt{4\frac{cntArea}{\pi}}$
	aspectRatio	ratio between the two bounding box axes	$\frac{axm}{axM}$
	extent	ratio between the convex hull area and the bounding box area	$\frac{cntArea}{bbArea}$
	solidity	solidity	$\frac{perimeter}{bbArea}$

Training and Validation: this task has for input the examples set consisting of the set of labelled RoIs, each one characterised by the corresponding image features. The output of this task is a binary image classifier that returns 1 if the RoI contains at least a fish specimen and 0 otherwise.

The learning process is aimed at selecting a minimal number of relevant features capable to maximize the margin between positive and negative examples. Details on the supervised Machine Learning approach for generating the binary classifier are given in Section 2.

Table S 2: The texture image features extracted from each single Region of Interest (RoI), where h represents the histogram of the grey level intensity of the image pixels and *std* the standard deviation.

**texture features**	*histIndex*	histogram shape index	$std\left(\frac{h}{\sum h}\right)$
	std	normalized grey standard deviation	$\frac{\sqrt{\sum_{i=0}^{255} x(i-\mu)^2}}{\mu}$, $\mu = \sum_{i=0}^{255} x_i,\ x \in \left(\frac{h}{\sum h}\right)$
	ent	entropy	$-\sum x \log(x),\ x \in \left(\frac{h}{\sum h}\right)$
	contrast	normalized interior/exterior contrast	$\frac{\lvert mean(gInt)-mean(gExt)\rvert}{mean(gInt)+mean(gExt)}$

1.2 Automated image recognition pipeline

The output of the training and validation process is a binary classifier ready to be used for the fish recognition of unknown images.

The automated image recognition pipeline shown in Figure S1b) is similar to the training and validation pipeline. The image content of the image acquired at time t is obtained by computing the difference with the image acquired at time $t-1$. During the automated recognition phase, no user interaction is needed and thus the relevant image features selected during the learning process are extracted from every identified RoI. Such image features are then applied to the binary classifier that returns 1 if the RoI contains at least a fish specimens, 0 otherwise.

2 Image recognition and feature selection

The Supervised Machine Learning approach used in this work is based on a Genetic Programming (GP) procedure [6, 11, 7], combined with a K-Fold Cross Validation as proposed in [4]. The GP-based software components were developed in Python by using the Pyevolve open source library [10].

GP is an evolutionary computation methodology capable of learning how to accomplish a given task. GP generates the solutions of the given task starting from an initial population of randomly generated mathematical expressions, based on a set of mathematical primitives, constants and variables. The initial

solutions are improved by mimicking the selection processes that occur naturally in biological systems through the Selection, Crossover and Mutation genetic operators [6].

In the used work, the binary classifiers evolved by the GP based approach are expressed as mathematical functions, whose variables correspond to the image features discussed in Section 1.

To evolve the GP-based classifiers the following parameters have to be chosen: the set of mathematical primitives, the number of individuals of the initial population, the number of generations the individuals evolve through, the specific parameters driving the crossover and the mutation among individuals, as shown in Table S3.

Table S 3: The GP parameters used for the evolution of the binary classifiers for the automated fish recognition task.

mathematical primitives	$\{+, -, *, /^*, sqrt^*, log^*, sin, cos, tan, atan\}$
variables	the image-features summarized in the Tables S1 and S2
constants	k random numbers from the range $[-10, 10]$,<br>where k is randomly selected in the range $[0, 10]$
initial population	ramped half-and-half
individual max depth	4
population size	500
max generations	200
raw fitness	the same proposed in [4]
scaled fitness	linear scaling
selector method	roulette wheel
crossover rate	0.9
mutation rate	$2 \cdot 10^{-4}$
elitism	true
termination criterion	max generations or raw fitness equals to 0.00

According to the feature selection method proposed in [9, 4], the relevant image features are identified by analysing the number of their occurrences among the classifiers of the population pool obtained by nesting the GP procedure within a K-fold Cross Validation framework (K = 10). Figure S3 shows the probability distribution of the image feature occurrences (green dotted line) according to the Bernoulli trial.

The red filled circles represent the number of occurrences of the image features in the population pool, while the vertical red lines represent the two-tails p-value equal to 0.001 used to select the relevant image features. Actually the image features on the right of the right vertical line are deemed as relevant.

From the relevance analysis, eight out of fourteen image features resulted relevant as shown in Table S4. Nevertheless, the automated image recognition was defined as an ensemble of all the individuals of the Population Pool containing the three most occurring image features (i.e. *contrast*, *equiDiameter*, and *ent*), as discussed in [4]. These individuals are listed in Table S5 and the

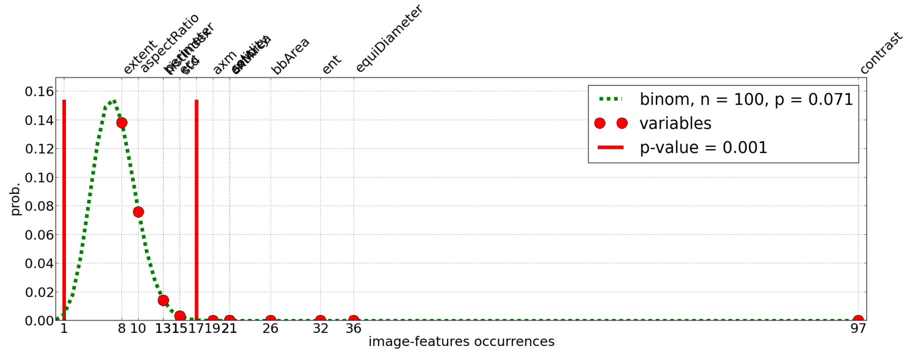

S 3: Relevance of the image features, according to the test statistics discussed in section [4, 9]. The abscissa represents the occurrences of the image features within the population pool. The ordinate represents the probability an image feature occurred in the population pool. The two red vertical lines represent the two-tails p-values with p equals to 0.001.

ensemble of the selected individuals is defined by the equation (Eq. S1):

$$\mathcal{E}(r) = \begin{cases} 1 & \text{if} \sum_{c \in C_{ens}} \overline{J_c(r)} > 0 \\ 0 & \text{otherwise} \end{cases} ,$$

$$\overline{J_c(x)} = \begin{cases} 1 & \text{if } eval(c(r)) > 0 \\ -1 & \text{otherwise} \end{cases} , \qquad \text{(Eq. S1)}$$

where r is the unknown RoI to be classified, C_{ens} is the set of individuals shown in Table S5 and $eval(c(r))$ is the real number obtained by evaluating the classifier c instantiating each variable with the corresponding image feature value.

3 Efficacy of the automated recognition for ecological analyses

This section contains the part of statistical analysis aimed at comparing the observed and recognised time-series that are not described in the main paper.

In particular, the PERMutaitonal Analisys Of VAriance (PERMANOVA) and the Generalised Linear Model (GLM) was performed and the corresponding results for values of bio-fouling scores grater than 0 are shown in the Tables S6 and S7, respectively.

References

[1] Opencv. `https://opencv.org/`. Accessed: 2017-11-20.

Table S 4: Relevant image features selected according to the methodology presented in [9], [4]. The left column shows the image feature, the mid column shows the number of image feature occurrences among the individuals of the Population Pool, the right column shows the p-value associated to the image feature. The image features in bold font are those used in the automated binary image classifier.

**image feature**	**Population Pool occurrences**	**p-value**
**contrast**	**97**	**8.68e-107**
**equiDiameter**	**36**	**1.09e-16**
**ent**	**32**	**2.32e-13**
bbArea	26	5.82e-09
solidity	21	6.85e-06
cntArea	21	6.85e-06
axM	21	6.85e-06
axm	19	7.86e-05

[2] Topological structural analysis of digitized binary images by border following. *Computer Vision, Graphics, and Image Processing*, 30(1):32 – 46, 1985.

[3] Christopher M. Bishop. *Pattern Recognition and Machine Learning (Information Science and Statistics).* Springer-Verlag New York, Inc., Secaucus, NJ, USA, 2006.

[4] Lorenzo Corgnati, Simone Marini, Luca Mazzei, Ennio Ottaviani, Stefano Aliani, Alessandra Conversi, and Annalisa Griffa. Looking inside the ocean: Toward an autonomous imaging system for monitoring gelatinous zooplankton. *Sensors*, 16(12), 2016.

[5] Geoff Dougherty. *Pattern Recognition and Classification: An Introduction.* Springer Publishing Company, Incorporated, 2012.

[6] John R. Koza. *Genetic Programming: On the Programming of Computers by Means of Natural Selection.* MIT Press, Cambridge, MA, USA, 1992.

[7] John R. Koza. Human-competitive results produced by genetic programming. *Genetic Programming and Evolvable Machines*, 11(3-4):251–284, September 2010.

[8] Thomas B. Moeslund. *Introduction to Video and Image Processing.* Springer London Dordrecht Heidelberg New York, 2012.

[9] Danny J. Papwortch, Simone Marini, and Alessandra Conversi. A novel, unbiased analysis approach for investigating population dynamics: A case

Table S 5: The individuals of the Population Pool that contain at least the three image features *contrast*, *equiDiameter*, and *ent*.

$sin\left(sin\left(\frac{ent}{solidity}\right)\right) \cdot \sqrt{log(contrast \cdot equiDiameter)}$
$log(tan(\sqrt{contrast})) + log(\sqrt{equiDiameter - ent})$
$\frac{ent+contrast}{ent+equiDiameter+(log(contrast)\cdot(ent+3.56))}$
$log\left(\frac{(ent-bbArea)\cdot contrast)}{-(3.88+equiDiameter)}\right)$
$log(\sqrt{cos(ent)} \cdot equiDiameter \cdot contrast)$
$log(equiDiameter \cdot contrast) + atan(sin(ent))$
$log((cos(equiDiameter) - ent - equiDiameter) \cdot contrast)$

study on *Calanus finmarchicus* and its decline in the north sea. *PLoS ONE*, 11(7):1–26, 07 2016.

[10] C. S. Perone. Pyevolve 0.6rc1. http://pyevolve.sourceforge.net/0_6rc1/. Accessed: 2017-12-01.

[11] Riccardo Poli, William B. Langdon, and Nicholas Freitag McPhee. *A field guide to genetic programming.* http://www.gp-field-guide.org.uk, 2008.

Table S 6: Main PERMANOVA test and pairwise comparisons (only significant variations are shown) of observed and recognized abundance data by month, regarding bio-fouling (F) grater than 0 and different combination of turbidity score (T). Numbers from 1 to 12 in the pairwise tests, indicated months from January (1) to December (12).

T0, F1	**observed**		**recognised**	
	**main test**			
	Pseudo-F10,117=5.74***		Pseudo-F10,117=5.40***	
	**pairwise**			
	Groups	t	Groups	t
	4, 5	2.13*		
	6,8	2.89*		
	8, 9	11.09***		
	10,11	2.64*		
T0, F3	**observed**		**recognised**	
	**main test**			
	Pseudo-F10,153=6.27***		Pseudo-F10,153=4.35***	
	**pairwise**			
	Groups	t	Groups	t
	4,5	2.13*		
	6,8	2.64*		
	8, 9	5.36***		
T3, F3	**observed**		**recognised**	
	**main test**			
	Pseudo-F10,379=11.67***		Pseudo-F10,379=13.56***	
	**pairwise**			
	Groups	t	Groups	t
	4, 5	2.04*	2, 3	3.34**
	8, 9	3.79***	10, 11	3.13**
	9, 10	3.05**	11, 12	3.82***

Table S 7: Results of GLM models for bio-fouling score (F) grater than 0, and several values of water turbidity (T). Chla_1mo = Chlorophyll-a concentration recorded by satellite one month before actual data; SST_sat = Sea surface temperature recorded by satellite; solar_irr = solar irradiance..

	Observed							Recognised						
**T0-F1**	NULL			117	457.83			NULL			117	131.57		
	Solar_irr	1	54.61	116	403.21	15.71***	neg	Chla_1mo	1	16.99	116	114.59	17.79***	neg
								SST_sat	1	4.75	115	109.84	4.97*	neg
	AIC=485.87	expl. Dev. =13.55%						AIC=334.41	expl. Dev.=19.79%					
**T0-F3**	NULL			153	487.73			NULL			153	164.46		
	Solar_irr	1	62.32	152	425.41	23.77***	neg	Chla_1mo	1	23.24	152	141.23	26.47***	neg
	SST_sat	1	14.4	151	411.02	5.49*	neg	SST_sat	1	8.67	151	132.56	9.87**	pos
	Chla_sim	1	17.69	150	393.33	6.75*	neg							
	AIC=591.44	expl. Dev. =19.35%						AIC=421.94	expl. Dev. =19.40%					
**T3-F3**	NULL			187	518.26			NULL			187	215.95		
	solar irr.	1	58.94	186	459.32	24.99***	neg	SST_sat	1	53.71	186	162.24	64.68***	pos
	wind speed	1	22.99	185	436.32	9.75**	neg	Chla_1mo	1	8.65	185	153.59	10.42**	neg
	AIC=699.81	expl. Dev. =15.81%						AIC=503.52	expl. dev.=28.88%					
